# In Situ Observation of Orientational Ordering in Polyimide Triboelectric Generators by Using Optical Second-Harmonic Generation Measurement

**DOI:** 10.1021/acs.jpclett.4c03044

**Published:** 2024-12-05

**Authors:** Mahato Maeda, Dai Taguchi, Takaaki Manaka

**Affiliations:** Department of Electrical and Electronic Engineering, Tokyo Institute of Technology, 2-12-1 O-okayama, Meguro-ku, Tokyo 152-8552, Japan

## Abstract

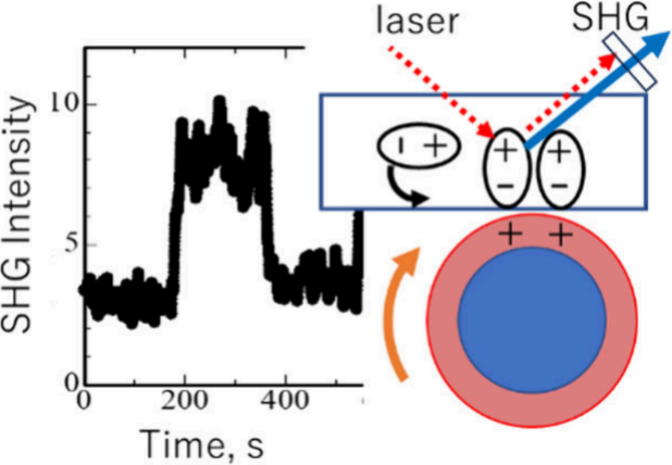

By using in situ optical second-harmonic generation (SHG) measurement, dynamical changing of polar molecular orientations caused by mechanical rubbing of polyimide triboelectric generators is observed. The polar orientational order is enhanced during rubbing, which relaxes with the time constant τ = 7.1 s. Analysis of s- and p-polarized SHG intensity under s-polarized laser incidence showed that molecules align along the rubbing direction with the tilt angle θ_0_ = 51° from the surface normal. By using the obtained time constant and the tilt angle, the output power in the *I*–*V* measurement is discussed. That is, the SHG result gives a theoretical maximum power of 2.0 μW/cm^2^, while the effective power observed in the *I*–*V* measurement is 0.26 μW/cm^2^, suggesting that merely part of produced power is received at external loads. The in situ SHG measurement gives helpful ways to discuss the power output of triboelectric generators using polar materials.

The generation of triboelectric monocharges, i.e., electrons and holes, has been recognized for long time. In recent years, the use of the monocharges as electrical power sources has developed with idea of emerging electrical generators such as triboelectric nanogenerators (TENGs).^1^ Accordingly, interest in innovative electronics applications, for example, self-powered sensors, interactive books, and other applications, is growing.^2−4^ Meanwhile, various types of TENGs are making it possible to convert heat, rain drops, and other energies in the surroundings to electrical power.^5,6^ By using advanced materials and processing techniques, the triboelectric monocharges are stably and efficiently created on a material’s surface.^7−9^ With effort to understand triboelectric monocharges,^10−12^ the triboelectric series table has been utilized as a helpful guide for enhancing triboelectric generators. However, the concept of triboelectric monocharges is not the whole story of the generation of electricity by friction.

Mechanical rubbing has been utilized to make an orientationally ordered state of surface molecular groups.^13,14^ As is well-known in the research and development (R&D) field of liquid crystal devices, mechanical rubbing gives easy and reliable ways to align molecular orientations.^15−17^ By using advanced measurement techniques, surface molecular orientations formed by rubbing have been investigated.^18−21^ Meanwhile, the understanding of molecular alignment mechanisms has been developed.^22−26^ For polar materials, the rubbing forces molecules to be orientationally ordered. This is a thermodynamically unstable high energy state, making a spontaneous transition to a low-energy isotropic state through dipolar depolarization. That is, if we focus on the orientationally ordered permanent dipoles, we can find new ways of generating triboelectricity. Accordingly, we reported the energy conversion mechanism through dipolar depolarization induced by rubbing;^27−30^ meanwhile, we showed that the electric-field-induced optical second-harmonic generation (EFISHG) measurement is useful to probe orientationally ordered molecular dipoles and the monocharges, one-by-one.^31−33^ By using the probe laser beam wavelength at 570 (SHG wavelength of 285 nm) and 1140 nm (SHG wavelength of 570 nm), we can probe, respectively, polar molecular orders and electrostatic charge generation by rubbing polyimides with cotton cloth. However, this situation is no longer sufficient to investigate power transmission phenomena in triboelectric generators. The dynamical changing of molecular orientational orders is the key that should be made clear by experiment. Electrostatic Kelvin probe measurements have been utilized to evaluate static charges.^34,35^ But in situ measurement is not an easy task, for the electrode probe and rubbing cloth must be positioned on the rubbed surface at the same time.

In this Letter, we report that the in situ SHG measurement gives a useful way to directly probe the dynamical change of molecular orientational orders induced during mechanical rubbing. The use of an optical laser probe makes it possible to arrange the film being rubbed mechanically on one side of the film; meanwhile, the orientational ordering is probed from the backside of the film. Results showed that the highly orientational ordered state of polyimide is induced, while it is disordered after the rubbing is stopped. Based on the SHG results, electrical power output in *I*–*V* measurements of triboelectric generators is discussed in terms of orientational ordering of polar molecules. It is noteworthy that the optical SHG measurements have been powerful tools to investigate polar orientational order in materials.^36^ However, it is not an easy task to identify whether surface ordering or the bulk contributes to the observed SHG signal,^37,38^ and optical simulations have been utilized to analyze the results. In the present study, by coupling the electrical measurement where displacement current depends on the portion of electrostatic polarization through the bulk, one can discuss the contribution of polar ordering through the film.

Figure 1 shows the optical arrangement used for in situ SHG measurements. A pulsed laser at a wavelength of 570 nm was used as the fundamental light (SHG wavelength, 285 nm) to probe the orientational ordering of the polar diphenyl ether group in polyimide.^31^ The laser pulses were s- and p-polarized light with vibrating electric fields  and , respectively, and was focused on the polyimide–cotton interface at the incident angle 45°. The s- or p-polarized SHG light in the reflected direction, which has electric field  or , respectively, is selected by using the analyzer, transmitted through the band-pass filters, and detected by using the photomultiplier tube. The cotton rubbing cloth was used to rub the polyimide surface. In more detail, the cotton cloth was attached to a rotatable Al bar, and the Al bar was revolved at 60 rpm by using a brushless motor. The laboratory coordinate system (*x*, *y*, *z*) was set as illustrated in Figure 1. That is, the sample surface is located in the *xy*-plane and the *z*-axis is the surface normal direction, and the rubbing was conducted along the *x*-direction.

**Figure 1:**
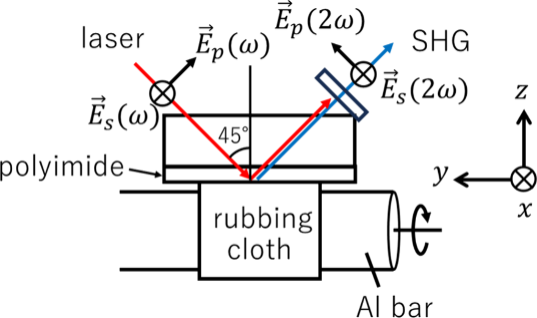
Optical arrangement for the in situ SHG measurement during rubbing.

Figure 2 shows the in situ SHG measurement results. The combination of the polarization of the laser beam and SHG lights is denoted as p-p, p-s, s-p, and s-s. For example, s-p denotes the combination of s-polarized laser beam and p-polarized SHG. The results showed that the SHG intensities in the p-p, s-p, and s-s combinations are mainly enhanced during rubbing. After the rubbing was stopped, the SHG intensity decreased. The results suggest that the rubbing forces molecular groups orientationally ordered in the rubbing direction, whereas the aligned molecules became disordered after the rubbing was stopped. Interestingly, the results showed that the ordering happens with time constant τ_1_ = 3.3 s, which is shorter than the time constant for the disordering process (τ_2_ = 7.1 s). This implies that the ordering process is forced mechanically by impact acting in a short time; meanwhile, the disordering process is governed by thermal relaxation through multiple collisions in rotational Brownian motion.

**Figure 2:**
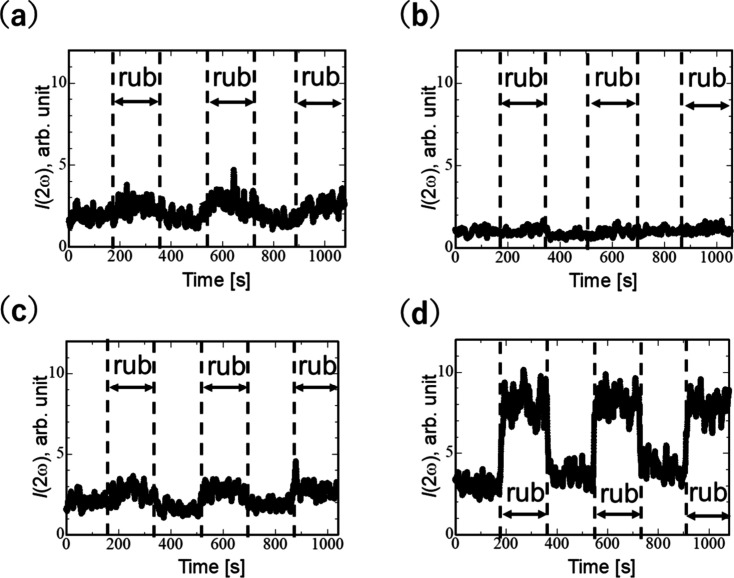
In situ SHG measurement during rubbing of polyimide with (a) p-p, (b) p-s, (c) s-p, and (d) s-s polarization conditions. The direction of s-polarization is pointing in the rubbing direction. The polyimide surface is rubbed for 170 s with interruption for 170 s.

It is instructive to compare the time constants with those expected using reported material parameters. Assuming that the thermal activation type relaxation time is  (τ_0_: pre-exponential factor, *H*: activation energy, *k*: Boltzmann constant, *T*: absolute temperature) and using the values *H* = 100 kJ/mol to fit τ^–1^ = 600 Hz at *T* = 410 K for the β-relaxation in ref (39), the relaxation time τ = 80 s is obtained at 300 K. The observed time constants are an order smaller than the calculation. This suggests that the relaxation time become smaller in the orientationally ordered state than in thermal equilibrium as the rotational motion is confined in an ordered state.^40,41^

To analyze the tilt angle of molecules induced by rubbing, as the first approximation, we assume that the rubbing orients the molecular dipoles with the tilt angle θ in *x*-*z* plane (*C*_*s*_-symmetry with the mirror plane in the *xz*-plane), and merely nonlinear molecular polarizability component β_*z*′*z*′*z*′_ along the *z*′-direction in the molecular coordinate system is nonzero. In more detail, taking into account the symmetry of the diphenyl ether group in polyimide, permanent dipoles point in the direction from the oxygen atom to the middle of the two benzene rings. The direction of the permanent dipole gives a *z*′-direction in the molecular coordinates, which is assumed to coincide with the direction of the induced dipole involved in the nonlinear optical transitions. Under the electric dipole approximation, the SHG intensities for the *C*_*s*_-symmetry are obtained as
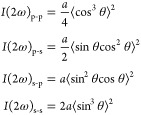
1where *a* is a constant and ⟨···⟩ represents average over molecules.^36^ If all molecules align along the *x*-direction (θ = π/2), *I*(2ω)_s-s_ ≠ 0 and *I*(2ω)_s-p_ = *I*(2ω)_p-s_ = *I*(2ω)_p-p_ = 0. As shown in Figure 2(d), *I*(2ω)_s-s_ dominates among the results for various polarization combinations. From the SHG intensity *I*(2ω)_s-s_ and *I*(2ω)_s-p_ during rubbing,  = 3.01 is obtained. Assuming that the molecules align in the same direction θ = θ_0_, the tilt angle θ_0_= 51° is obtained. Note that the modulation of light intensity due to optical arrangement is effectively represented by constant *a* in eq 1. By taking the ratio between *I*(2ω)_s-s_ and *I*(2ω)_s-p_, the effect of the optical arrangement is canceled to calculate tilt angles. It is also noteworthy that the mechanical rubbing has been also reported to permanently align molecules at polyimide surface.^18^ In Figure 2(d), the SHG intensity in the interval between the rubbings increased step-by-step. The in situ SHG measurement clearly shows that the orientational order is highly enhanced during rubbing in comparison with the permanent alignment of molecules that remained after the rubbing was stopped.

Figure 3 shows the *I*–*V* measurement results with and without a rubbing polyimide/ITO device. By linearly approximating *I*–*V* curve, short-circuit current and open-circuit voltage are obtained as *I*_s_= +11.5 nA and *V*_oc_= −92 V, respectively, which results in the maximum power  μW/cm^2^ (*A*: rubbing area). The power output is discussed using SHG results. Based on the model for triboelectric generators through dipolar depolarization, the short-circuit current , open-circuit voltage , and maximum power  are expected (*P*_0_: initial polarization, τ: dipolar relaxation time, *C*_s_: static capacitance).^30^ Assuming that the permanent dipoles point in the direction θ_0_ = 51° as the SHG measurement showed, the initial polarization *P*_0_=*N*μcos θ_0_ is estimated as 6.0 × 10^–7^ C/cm^2^ (*N*: density of monomer unit, μ: permanent dipole moment), where *N* = 2.2 × 10^27^ m^–3^ was calculated from lattice constants reported in ref (42) and μ = 1.3 D was obtained using a semiempirical quantum mechanical calculation. Using the initial polarization *P*_0_ and *C*_s_= 6.4 nF with rubbing area *A* = 1 cm^2^, the open-circuit voltage is estimated as  = −94 V, agreeing with the *I*–*V* results. The time constant τ_2_ = 7.1 s and the initial polarization *P*_0_ show that the short circuit current is  = 85 nA. That is, the short-circuit current from the *I*–*V* is 14% of that expected based on the SHG results, suggesting that a part of current is presumably lost due to the leakage path. As a total, the maximum power *P*_SHG_ = 2.0 μW/cm^2^ is expected to be transmitted based on the SHG measurement, but the *I*–*V* measurement showed that merely 13% of the power is effectively received at external loads. It is noteworthy that we discussed the electrical power output assuming that the permanent dipoles are orientationally ordered in the bulk. This is in contrast with the idea that merely surface molecular groups are permanently ordered in the rubbed polyimide layer for liquid crystal devices.^18^ The present discussion on electrical power transmission in combination with the SHG measurements points out that the mechanical rubbing presumably induces polar orientational ordering through the bulk of the film.

**Figure 3:**
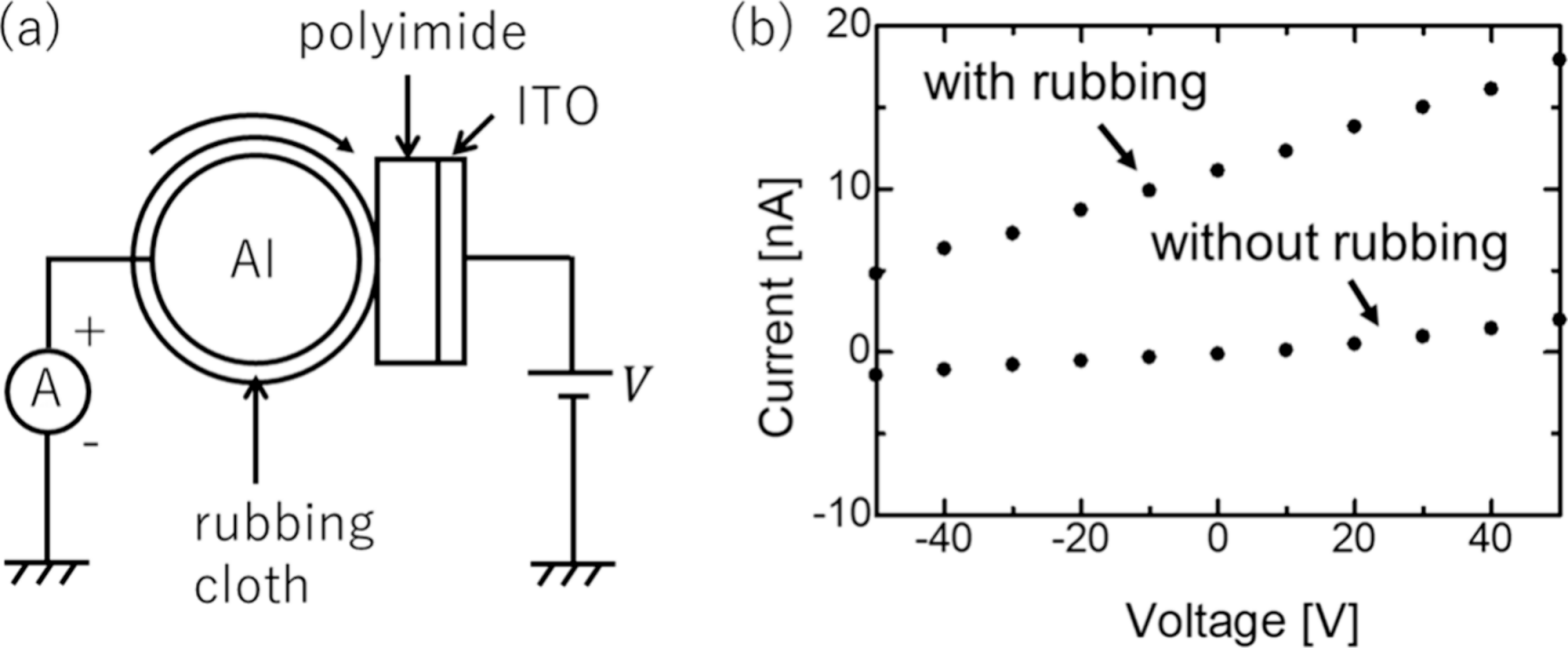
(a) Experimental setup for *I*–*V* measurement during rubbing. (b) The *I*–*V* results of PMDA-ODA polyimide/ITO triboelectric generators with rubbing (Δ*N* = 2 mN·m, 60 rpm) and without rubbing (0 rpm).

It will be worth discussing the polar ordering process based on the in situ SHG results. The enhancement of SHG signals during rubbing showed the polar orientational ordering of molecular groups in the main chain, giving the idea that the rubbing cloth touches the polyimide molecule and drags along the rubbing direction to orientationally align polymer chains. This ordering is characterized by a highly ordered state that likely emerges into the bulk. Accordingly, the mechanically induced orientation at the rubbed surface must cooperatively induce the polar orientational order under the surface. In analogy to the spontaneous polarization in ferroelectric materials, one possible scenario is that the orientationally ordered permanent dipoles at the surface produce a local electric field that enhances orientational ordering in the bulk. Unlike the ferroelectric polymer, the enhanced ordered state is not in equilibrium and spontaneously makes the transition to an isotropic state through rotational Brownian motion after the rubbing stops.

In summary, an in situ SHG measurement was conducted to study dynamical polar orientational orderings that are the origin of electrical power transmission through dipolar depolarization. In experiments, polyimide triboelectric generators were used where permanent dipoles were considered to be active for electric power transmission. Results showed that a highly ordered state is induced during rubbing, which relaxes after the rubbing is stopped. By using the tilt angle during rubbing and relaxation time extracted from the in situ SHG measurements, the electrical power output of triboelectric generators in *I*–*V* measurements was discussed in terms of the molecules’ orientational ordering. The in situ SHG measurements are helpful to discuss the dynamical change of molecular orientational ordering that is a key to electrical power transmission through dipolar depolarization.

## Experimental Methods

The polyimide/silica device was used in SHG measurements. The pyromellitic dianhydride (PMDA)-4,4′-oxydianiline (ODA) polyamic acid (PAA) was dissolved in *N*,*N′*-dimetylacetoamide (DMAc) at 10 wt %. The silica substrates with the area 25 × 25 mm were cleaned by using a UV/ozone cleaning apparatus in an oxygen atmosphere, and the solution was spread onto the substrates using spin-coating (1500 rpm, 30 s). Subsequently, the PAA/silica samples were placed in an oven at 300 °C for 1 h to thermally imidize the PAA in a dry nitrogen atmosphere. The resulting samples were used as polyimide/silica devices, where the polar diphenyl ether group with nonzero permanent dipole is able to transmit electric power through dipolar depolarization. For *I*–*V* measurements, polyimide/ITO devices were prepared in a way similar to the polyimide/silica devices. In *I*–*V* measurements, the voltage source was connected to the ITO electrode, and the electroammeter was connected to the cotton rubbing cloth through the Al bar.

In SHG and *I*–*V* measurements, the cotton cloth rubs the polyimide surface with an area *A* = 1.0 cm^2^, where the torque on the rotating Al bar (60 rpm) is increased by Δ*N* = 2 mN·m. In more detail, the substrate was fixed on a stage and moved toward the rotating rubbing cloth by using a stepping-motor that is capable of positioning with 1 μm step. Meanwhile the torque on the rotating Al bar is continuously measured. Accordingly, the stage position is fixed where the torque increased by Δ*N* = 2 mN·m in reference to that measured without touching the rubbing cloth. The rotation speed is kept constant by using a speed-controlled brushless motor. The diameter of Al bar is 25 mm, and the cotton cloth rubs the polyimide surface at 7.9 cm/s. The measurements are conducted in the ambient laboratory.
